# Temporal patterns of wildlife roadkill in the UK

**DOI:** 10.1371/journal.pone.0258083

**Published:** 2021-10-06

**Authors:** Sarah Raymond, Amy L. W. Schwartz, Robert J. Thomas, Elizabeth Chadwick, Sarah E. Perkins

**Affiliations:** 1 Cardiff School of Biosciences, Cardiff University, Cardiff, United Kingdom; 2 Eco-explore Community Interest Company www.eco-explore.co.uk, Cardiff, United Kingdom; Universita degli Studi di Sassari, ITALY

## Abstract

Wildlife-vehicle collisions are one of the main causes of mortality for wild mammals and birds in the UK. Here, using a dataset of 54,000+ records collated by a citizen science roadkill recording scheme between 2014–2019, we analyse and present temporal patterns of wildlife roadkill of the 19 most commonly reported taxa in the UK (84% of all reported roadkill). Most taxa (13 out of 19) showed significant and consistent seasonal variations in road mortality and fitted one of two seasonal patterns; bimodal or unimodal: only three species (red fox *Vulpes vulpes*, European polecat *Mustela putorius* and Reeves’ muntjac deer *Muntiacus reevesi*) showed no significant seasonality. Species that increase movement in spring and autumn potentially have bimodal patterns in roadkill due to the increase in mate-searching and juvenile dispersal during these respective time periods (e.g. European badger *Meles meles*). Unimodal patterns likely represent increased mortality due to a single short pulse in activity associated with breeding (e.g. birds) or foraging (e.g. grey squirrels *Sciurus carolinensis* in autumn). Importantly, these patterns also indicate periods of increased risk for drivers, potentially posing a greater threat to human welfare. In addition to behaviour-driven annual patterns, abiotic factors (temperature and rainfall) explained some variance in roadkill. Notably, high rainfall was associated with decreased observations of two bird taxa (gulls and Eurasian magpies *Pica pica*) and European rabbit *Oryctolagus cuniculus*. By quantifying seasonal patterns in roadkill, we highlight a significant anthropogenic impact on wild species, which is important in relation to conservation, animal welfare, and human safety.

## Introduction

Roads represent a significant source of mortality for wildlife. In the UK, for example, collisions with vehicles are the leading observed cause of mortality of barn owls (*Tyto alba*) [1], European badgers (*Meles meles*) [2], and Eurasian otters (*Lutra lutra*) [3]. The impact of UK roads on wildlife has likely increased over the last 50 years due to expansion of road infrastructure and an increase in vehicle use. For example, there were 4.2 million vehicles on the UK’s roads in 1951, compared with 37.3 million by the end of 2016 [4], and over the same period, the overall length of the road network increased from 184,000 to 246,500 miles [5]. National estimates of annual wildlife mortality due to roads in the UK include 50,000 badgers [2], and between 167,000 and 335,000 European hedgehogs (*Erinaceus europaeus*), representing 10–20% of the latter species’ annual mortality [6]. At least 12,000 deer are estimated to be killed on roads in Scotland alone each year [7]. Up to 13% of captive-bred ring-necked pheasants (*Phasianus colchicus*) die due to collisions with cars following their release into the wild [8]; with an estimated 35–50 million pheasants released as quarry for recreational shooting yearly [9, 10], this implies that 4.5–6.5 million pheasants die on roads in the UK each year. The scale of the problem is clearly important from road traffic safety and animal welfare perspectives and understanding temporal variation (within and between years) may help with planning mitigation [11, 12]. Roadkill also represents an important food resource for scavengers and “meso-predators” [10, 13], and by helping to maintain high numbers of the latter, roadkill may have knock-on implications for prey populations [10].

Previous studies (on small vertebrates in Canada [14]; birds in Europe [15]; and vertebrates in Belgium [16]) have shown that periods of searching for mates or increased foraging activity can lead to peaks in road mortality. Likewise, juvenile dispersal is likely to influence seasonality in road mortality, due to an increased abundance of free-ranging but inexperienced juveniles [14, 15, 17, 18]. Interestingly, there is evidence for strategic behavioural responses to roads, with some bird species exhibiting learned avoidance behaviours, specifically by adjusting flight initiation distance depending on expected vehicle speed [19]. Increased seasonal mortality among birds might therefore be expected at fledging times, due to a lack of experience or learnt behaviours among younger individuals. To date, the majority of studies looking at temporal patterns in roadkill have focused on individual species [20–22] or more broadly, on ungulate species because of the substantial threat that they pose to human safety when involved in wildlife vehicle collisions [23, 24].

Seasonal variation in roadkill has also been linked to environmental variables; amphibian roadkill, for example, increases with rainfall events and higher humidity, due to increased activity in wet conditions [11, 14, 25], and road mortality of Eurasian otters is positively associated with increased river flow—an increase in water levels can cause otters to cross roads rather than utilising channels underneath [3]. Conversely, increased humidity has been negatively associated with avian roadkill [11]. High temperatures have been linked to high numbers of roadkill of many vertebrates, including mammals, amphibians, and reptiles [11, 26]; reptiles may be particularly at risk due to their attraction to warm road surfaces.

A large proportion of roadkill data are collated via citizen science schemes, many of which are opportunistic (or *ad-hoc*) records [27], as is the case with the current study. Although previous studies examining temporal patterns in roadkill have traditionally used standardised counts along a given road transect [see e.g. 3, 11, 16, 28–32], such targeted studies are costly in both time and finances, and are limited in the area that can be covered, due to logistical constraints [33]. The use of citizen science allows large amounts of roadkill data to be collected over a broad geographic and time span [34–36]. Inherent with data of this type are limitations in data quality, including potential misidentification of species [37], searcher efficiency [38, 39], and heterogenous distribution of reporting over space and time [40]. Additionally, presence-only data often do not have a fixed sampling protocol. Due to the large size of many citizen science datasets, however, the ratio of signal to noise is favourable, and analysis can identify clearly detectable patterns [41]. Volunteer-based recording schemes usually provide reliable data, and the increased sampling effort (and therefore greater statistical power) due to the potentially large number of participants can be advantageous when compared to traditional survey methods [42, 43].

In this study, we analysed over 54,000 volunteer-generated records to examine temporal patterns of wildlife roadkill in the UK. Using this large dataset, we identified the seasonal variations in road mortality of wildlife in the UK and discussed the behavioural, ecological and abiotic factors that may have driven this variation as well as the utility of the data for mitigation. Specifically, we aimed to determine: 1) whether there is seasonal variation in roadkill and 2) whether seasonality in roadkill is taxon-specific. We also 3) tested for the influences of unseasonal temperature and rainfall on taxon-specific temporal variation.

## Methods

### Roadkill data

The data used for this study were compiled by a citizen science roadkill recording scheme hosted at Cardiff University (www.projectsplatter.co.uk) spanning 2014–2019. Data constituted *ad hoc* records of wildlife roadkill, comprising date, location and species identity, submitted year-round and UK-wide by citizen scientists, and by other organisations (e.g. local authorities and species interest groups, such as Cardiff University Otter Project https://www.cardiff.ac.uk/otter-project). Basic validation checks were made by ensuring that records were within a species’ known geographic range and mapped onto a road location. Previous studies on species identification skills by participants in other citizen science roadkill recording schemes have shown data collection to be very reliable [33, 44]. Despite this, we acknowledge that identification skills of some participants, and the condition of carcasses, may not enable all roadkill to be identified to species level, and have grouped some taxa accordingly (see below–‘Taxa included in this study’). Dates, locations, and species identification of roadkill were collected for 5 years from January 1^st^ 2014 until December 31^st^ 2019, forming a dataset of ca. 54,000 records, with the average number of individual reporters to the project each year being around 562 (see S1 Text and S1–S3 Figs for a summary of reporter behaviour). The data used for this study are available online via the National Biodiversity Network Atlas (https://registry.nbnatlas.org/public/show/dp205).

### Seasonal variation in wildlife roadkill in the UK

#### Taxa included in this study

Taxa for which species-level identification was not frequently reported were grouped. Specifically, all gull species (Laridae) were grouped as ‘gulls’; brown hare (*Lepus europaeus*) and mountain hare (*Lepus timidus*) were grouped as ‘hares’. Roadkill reported as ‘rat’ or ‘squirrel’ (i.e. without a species-level identity) was assumed to be brown rat (*Rattus norvegicus*) or grey squirrel (*Sciurus carolinensis*) respectively, due to the relative rarity of black rat (*Rattus rattus*) and red squirrel (*Sciurus vulgaris*) in the UK. For analysis of seasonal variation in total roadkill, all species/taxa (hereafter referred to as taxa/taxon) were included. For taxon-specific analyses, taxonomic groups with fewer than fifty records in any given year were excluded. The resulting dataset included 11 taxa of mammals (‘hare’, brown rat, grey squirrel, European badger, roe deer *Capreolus capreolus*, Reeves’ muntjac deer *Muntiacus reevesi*, red fox *Vulpes vulpes*, European hedgehog, Eurasian otter, European polecat *Mustela putorius*, and European rabbit *Oryctolagus cuniculus*), and 8 taxa of birds (‘gull’, barn owl, European blackbird *Turdus merula*, common buzzard *Buteo buteo*, Eurasian magpie *Pica pica*, ring-necked pheasant, tawny owl *Strix aluco*, and common woodpigeon *Columba palumbus*).

#### Modelling of seasonal variation

For each taxon, we examined seasonal variation in the occurrence of roadkill. To facilitate direct comparisons between taxa and overcome any inter-year biases in number of animals reported, i.e. the inherent bias in data that is collected *ad hoc*, monthly totals for each taxon were scaled as a percentage of the given taxon’s annual total records for that year (to the nearest 1%), as has been done in previous studies [e.g. 17]. Due to a substantial increase in the number of reports occurring in July and August 2019 as a result of national media coverage of the project (approximately 3x and 2x the mean number of reports recorded in July and August across 2014–2019, respectively), records for these months were subsampled to reduce the numbers of records by a third, and a half, respectively. Subsampling of the original dataset for these months was randomised and repeated 10,000 times, and the mean count of each taxon was taken from across the subsamples and used in the analyses. Each taxon was analysed using a separate General Additive Model (GAM) with the monthly percentage of roadkill records in each given year as the dependent variable. A Poisson error family was used with the most suitable link function (log, identity or square-root), based on which produced the lowest Akaike Information Criterion (AIC) value (except where high dispersion resulted in poor model fit, identified by an over-dispersion statistic value >2.0, in which cases we used a negative binomial family, and log-link function) [45]. To enable different seasonal patterns to be modelled for each year, month was treated as a numerical independent variable, spanning the period from month 1 (January 2014) to month 72 (December 2019), modelled as a non-parametric smoother using a thin-plate regression spline, implemented in the “mgcv” package in R [46] (using a maximum k-value of 36, chosen as being half of the number of months in the time-series).

#### Modelling abiotic factors

**Fig 1:**
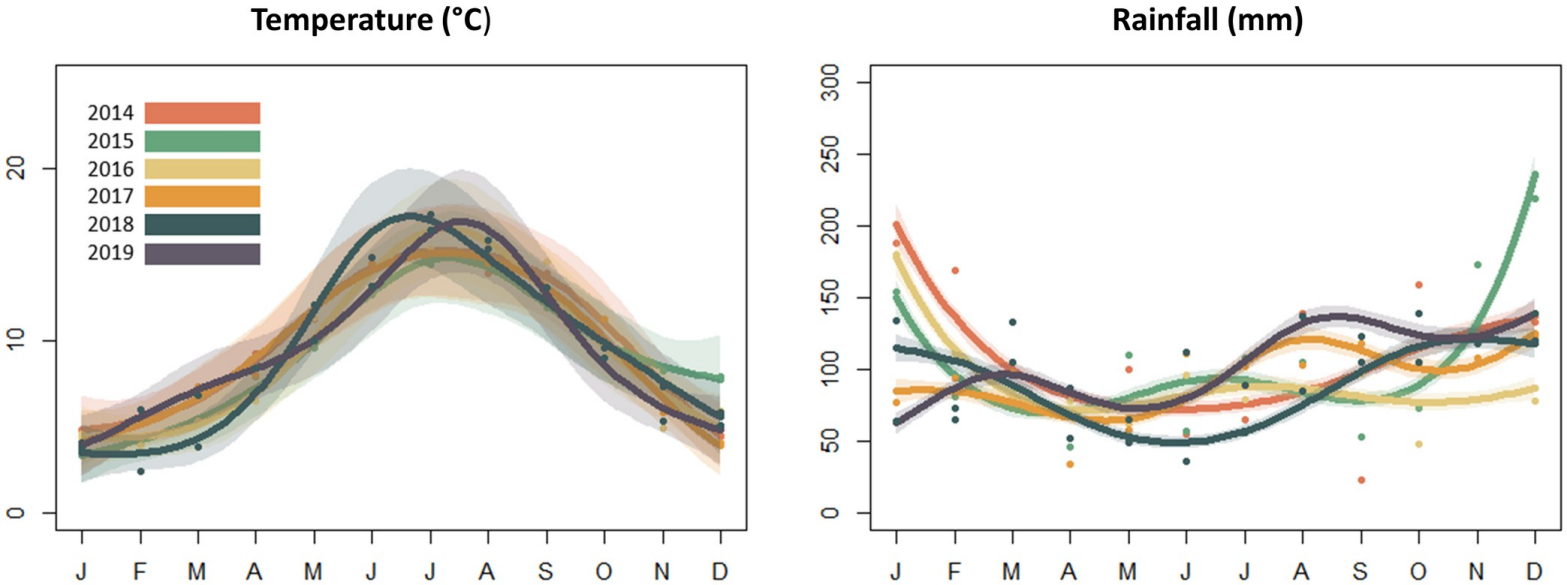
Variations in temperature and rainfall. Smoothed plots showing variation in mean temperature and rainfall across the UK during the six years that we report roadkill data from; 2014–2019.

To examine the relative importance of possible drivers of inter-annual variation in the seasonal pattern of roadkill of different taxa and to control for the potential impact of seasonal changes in weather on roadkill, we collated UK monthly mean temperature and rainfall data from the Meteorological Office (https://www.metoffice.gov.uk/climate/uk/summaries/datasets; Fig 1). Using these data, we fitted a GAM to the number of roadkill reports per month for each taxon, fitted as before i.e. using a Poisson error family (and the most suitable link function) except where high dispersion resulted in poor model fit, as described above (Table 1; [47]). As with the species-specific models of seasonal variation described above, month was treated as a numerical independent variable, spanning the period from month 1 (January 2014) to month 72 (December 2019), modelled as a non-parametric smoother using a thin-plate regression spline, with a maximum k-value of 36). This analysis therefore tested whether there were linear effects of temperature and/or rainfall that explained additional variation in roadkill, while controlling for the overall non-linear seasonal pattern of variation in roadkill, e.g. whether a particularly wet period influenced the number of a given taxon observed as roadkill, compared to the seasonal expectation.

**Table 1:** Summary table of statistical values (Chi sq. and p-value) for Generalised Additive Models examining relationships between seasonal roadkill patterns and two abiotic variables: Mean temperature and rainfall.

	Mean Temperature		Rainfall		Model family
Taxon	Chi. sq	p-value	Chi. Sq	p-value	
All taxa	0.072	0.789	1.14	0.286	Poisson
Badgers	1.014	0.314	0.202	0.653	Poisson
Foxes	0.328	0.567	1.627	0.202	Poisson
Otters	1.753	0.186	0.612	0.434	Poisson
Polecats	1.798	0.18	0.034	0.853	Neg. binomial
Brown Rats	0.001	0.973	0.54	0.817	Poisson
Grey Squirrels	0.015	0.902	1.797	0.18	Poisson
Hares	3.064	0.08	0.518	0.472	Poisson
Rabbits	0.4	0.527	*4*.*37*	*0*.*037*	Poisson
Hedgehogs	0.004	0.949	0.767	0.381	Poisson
Barn Owls	0.019	0.891	0.449	0.503	Neg. binomial
Tawny Owls	0.227	0.633	1.452	0.228	Neg. binomial
Buzzards	0.236	0.627	0.038	0.846	Neg. binomial
Pheasants	0.419	0.517	0.12	0.729	Poisson
Gulls	2.931	0.087	*12*.*003*	*<0*.*001*	Poisson
Woodpigeons	0.055	0.815	0.195	0.659	Poisson
Magpies	0.041	0.839	*9*.*173*	*0*.*002*	Neg. binomial
Blackbirds	0.199	0.656	2.899	0.089	Neg. binomial
Muntjac Deer	0.22	0.639	0.269	0.604	Neg. binomial
Roe Deer	0.206	0.65	0.044	0.833	Neg. binomial

Statistically significant (and marginal) values are shaded; italics indicate a significant negative relationship, bold a significant positive relationship. In all instances, the degrees of freedom were 1.

## Results

### Seasonal variation in overall abundance of roadkill

**Fig 2:**
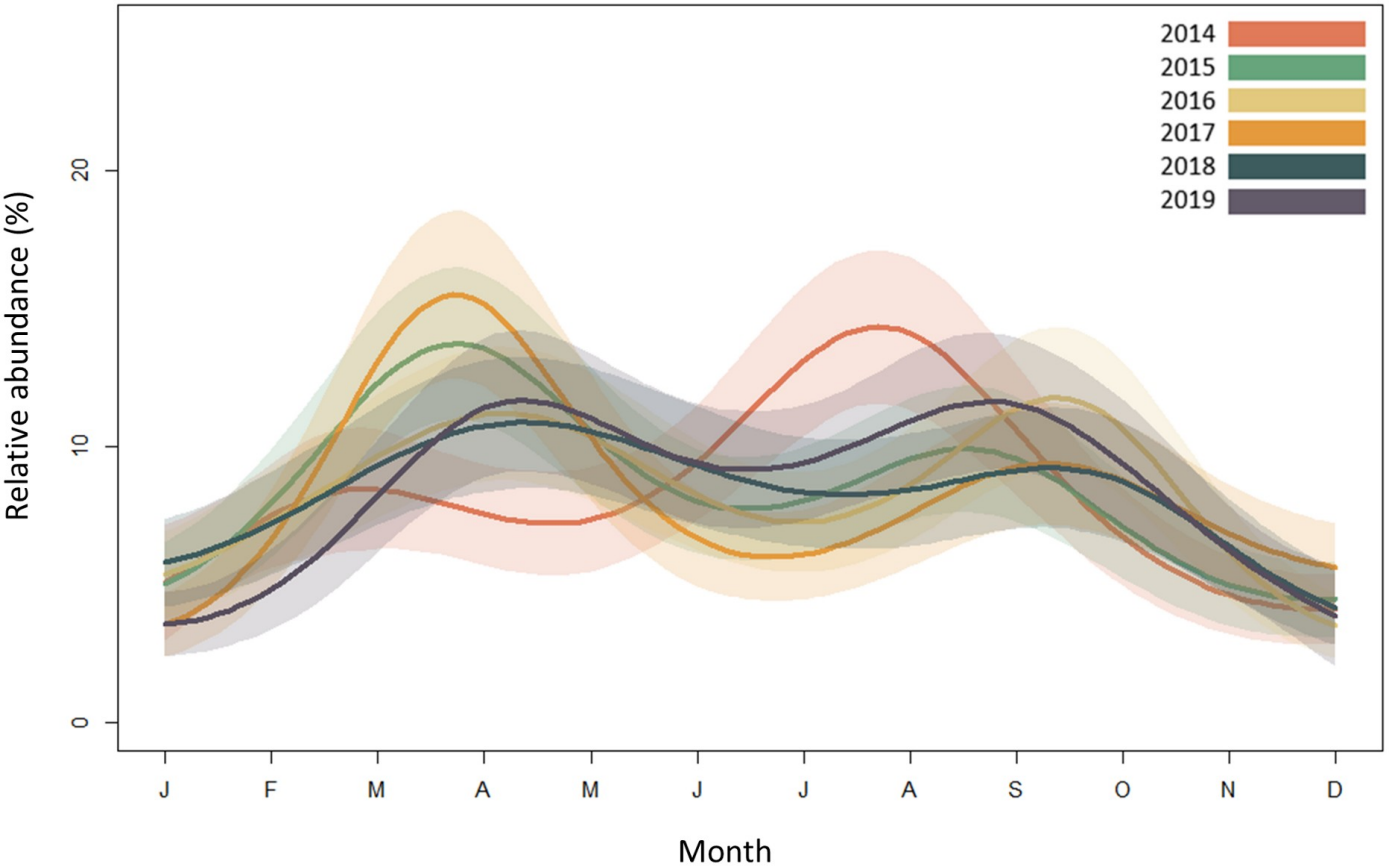
Seasonal variation in vertebrate roadkill in the UK. Abundance is quantified as the percentage of total number of roadkill reports per year, across all taxa. Records span 6 years, from 1^st^ January 2014 to 31^st^ December 2019. Shaded areas show 95% confidence intervals.

The total number of animals reported as roadkill varied significantly across the annual cycle in all years (e.d.f. = 35, Chi sq = 59.8, *p* = 0.006). Although seasonal variation in roadkill differed between years, typically there were fewer records during winter, and a peak in spring / summer (Fig 2).

### Seasonal variation in roadkill of mammals

**Fig 3:**
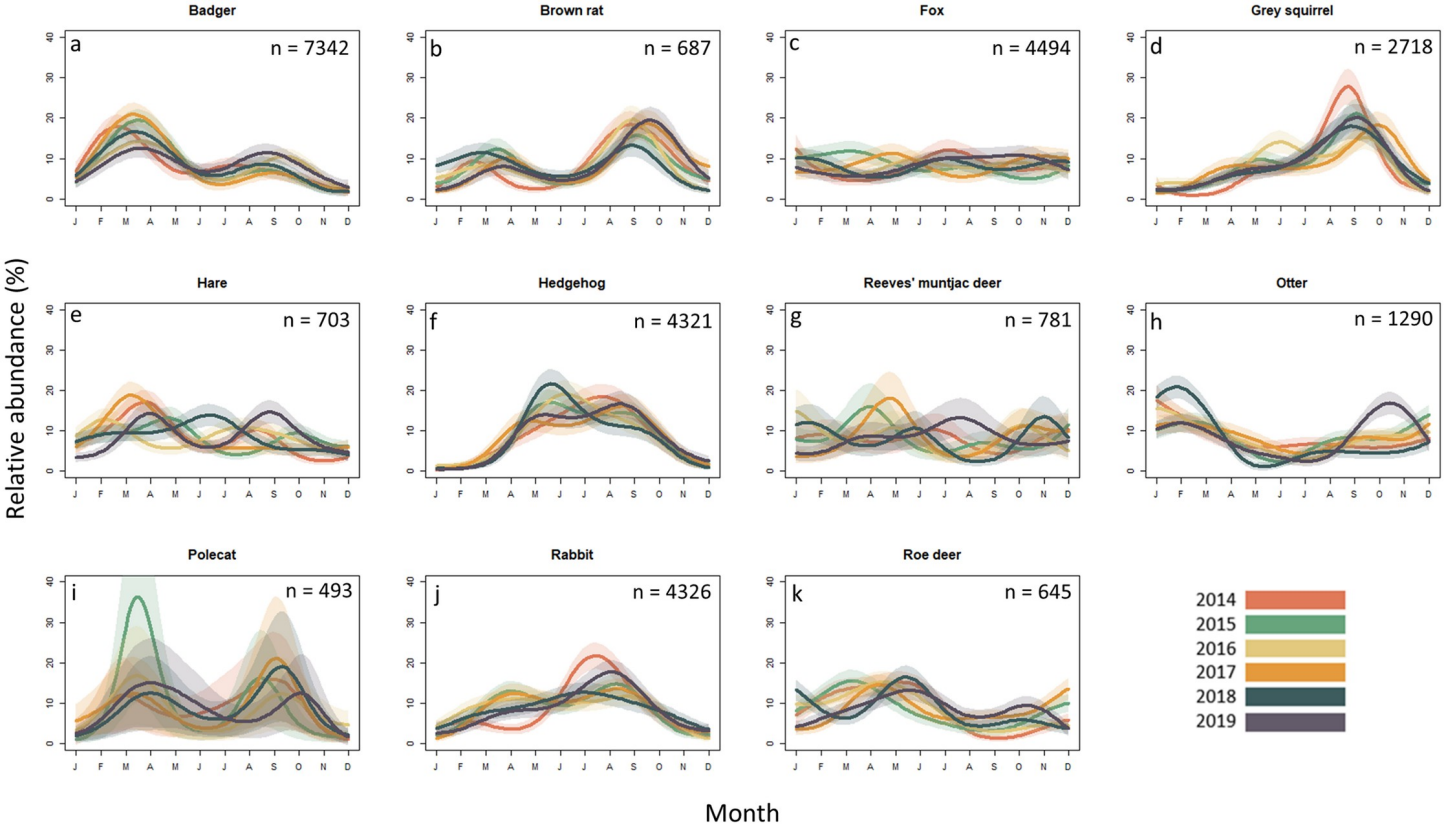
Seasonal variation in roadkill of mammals. Abundance (monthly percentage of annual total for each taxon) of the most common roadkill wild mammals in the UK. Records span 1^st^ January 2014 to 31^st^ December 2019. Shaded areas show 95% confidence intervals.

Seasonal variation was statistically significant for all but three mammal species, including red fox (which showed no seasonality), Reeves’ muntjac deer and polecats (both of which appeared to show seasonality in some years, but with relatively inconsistent patterns). Two broad patterns were apparent across the remaining species: bimodal and unimodal (Fig 3). Badger roadkill records showed a main peak in late winter-early spring (February to April), with a smaller autumn peak in September to October (e.d.f. = 35, Chi sq. = 173, *p* <0.001), although 2019 showed a marked and unusual late summer peak in mortality. Across all five years, December had the fewest reports of roadkill badgers (Fig 3a). Roe deer mortality showed a similar, but less marked seasonality (e.d.f. = 35, Chi sq. = 141.5, *p* <0.001, Fig 3k), with a larger spring peak, and a smaller secondary peak in autumn exhibited during 2018 and 2019. For brown rats, the highly consistent bimodal pattern differed from that for badgers, with the main peak in autumn and a smaller peak in spring (e.d.f. = 35, Chi sq. = 153.4, *p* <0.001, Fig 3b). Peaks were seen for hares in spring, and in late summer in most years, although for 2018 the pattern is inconsistent with this (e.d.f. = 35, Chi sq. = 92.4, *p* <0.001, Fig 3e).

Otter was the only mammal species to exhibit a winter peak in roadkill records (e.d.f. = 35, Chi sq. = 150.2, *p* <0.001, Fig 3). Other taxa showing single peaks in records include hedgehogs in summer (e.d.f. = 35, Chi sq. = 216, *p* <0.001, Fig 3f), rabbits and grey squirrels in summer-autumn (rabbits peaking in August, e.d.f. = 35, Chi sq. = 183.3, *p* <0.001, Fig 3j) and squirrels peaking consistently in September each year; e.d.f. = 35, Chi sq. = 222.5, *p* <0.001, Fig 3d). There was no statistically significant variation in the monthly reporting of foxes (e.d.f. = 35, Chi sq. = 31.6, *p* = 0.635, Fig 3c) or muntjac deer (e.d.f. = 35, Chi aq. = 30.78, *p* = 0.672). Although polecats appeared to exhibit bimodal peaks in spring and autumn, there was a large amount of variability (and smaller sample size than other mammals) and this apparent seasonality was not found to be statistically significant (e.d.f. = 35, Chi sq. = 29, *p* = 0.751, Fig 3i).

### Seasonal variation in roadkill of birds

**Fig 4:**
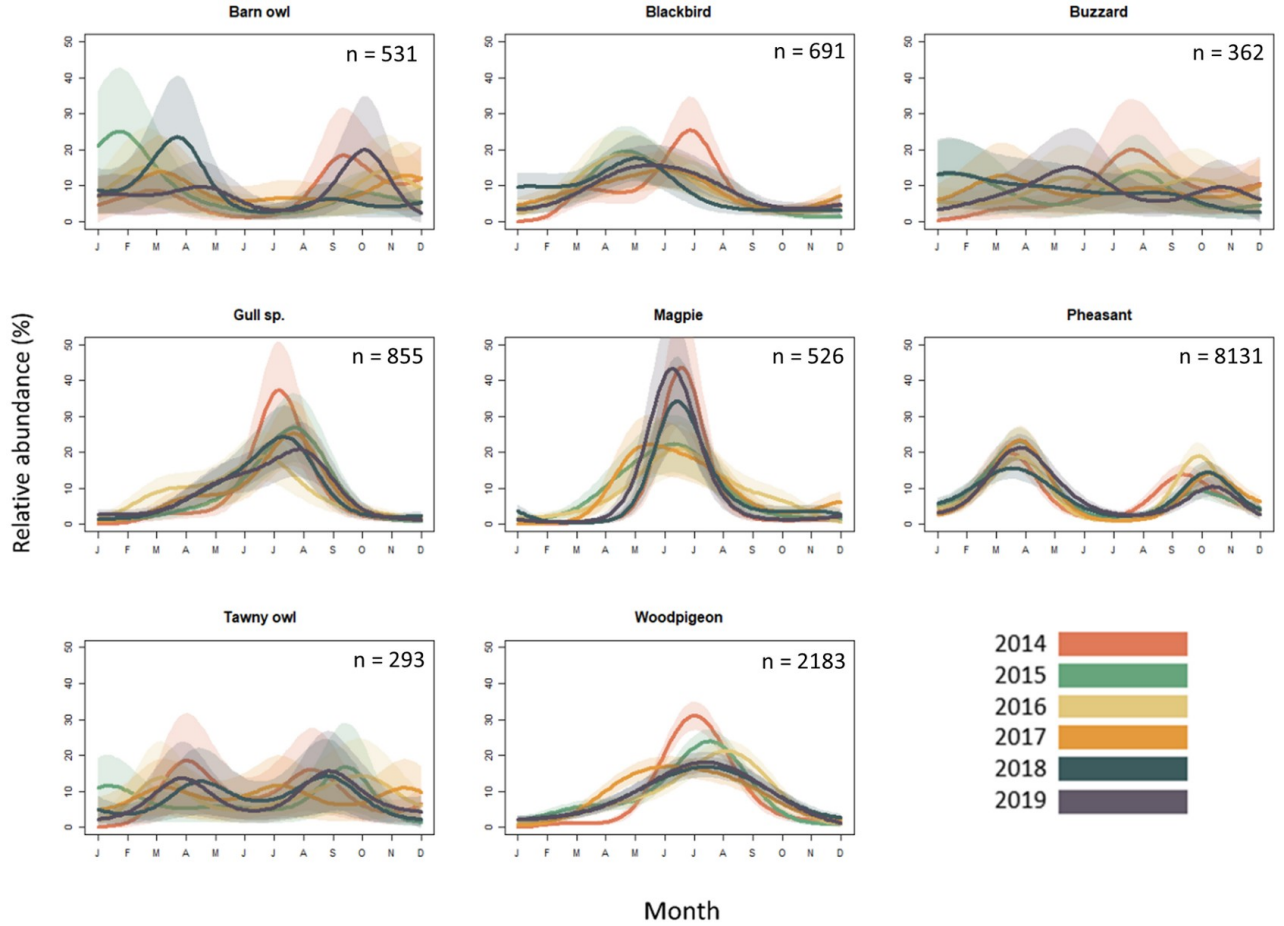
Seasonal variation in roadkill of birds. Abundance (monthly percentage of annual total for each taxon) of the most commonly reported roadkill wild birds in the UK. Records span 1^st^ January 2014 to 31^st^ December 2019. Shaded areas show 95% confidence intervals.

The predatory birds in the present study (barn owl, tawny owl, and common buzzard) showed highly variable patterns of seasonal variation (Fig 4a, 4c and 4g). Notably, the high between-year variance in temporal dynamics of the three predatory bird taxa is in sharp contrast with the non-predatory bird taxa, which have much more consistent patterns of seasonal variation. Within-year temporal variation in barn owl, tawny owl and common buzzard roadkill reports was non-significant for each species (e.d.f. = 35, Chi sq. = 22.41, *p* = 0.951; e.d.f. = 35, Chi sq. = 18.3, *p* = 0.991; e.d.f. = 35, Chi sq. = 14.86, *p* = 0.992, respectively) (Fig 4a, 4c and 4g).

Pheasants showed a strongly bimodal seasonal distribution in roadkill reports (e.d.f. = 35, Chi sq. = 225.7, *p* <0.001, Fig 4f). The first and most prominent peak occurred in spring, around March/April, followed by a lull during the summer, and another smaller but distinct peak during the autumn. This bimodal pattern of pheasant roadkill reports was consistent across all six years of the study. Half of the eight bird taxa showed a single, statistically significant peak in roadkill records, occurring during the spring and summer months (Fig 4b, 4d, 4e and 4h). The highest number of reports of gulls usually occurred in July (e.d.f. = 35, Chi sq. = 153.4, *p* <0.001, Fig 4d), woodpigeon roadkill reports peaked in the summer months of June/July/August (e.d.f. = 35, Chi sq. = 448.4, *p* <0.001, Fig 4h), and magpie roadkill peaked in June or July (e.d.f. = 35, Chi sq. = 190, *p* <0.001, Fig 4e). Blackbirds were reported abundantly as roadkill throughout the spring and summer (e.d.f. = 35, Chi sq. = 84.96, *p* <0.001). In 2015–2018, blackbird roadkill usually peaked in May, whereas the peak was shifted to July in 2014 and 2019 (Fig 4b).

### Abiotic factors

Seasonal variation in mean air temperatures was highly consistent between years 2014–2019, whereas the seasonal pattern of mean monthly rainfall was much more variable (see Fig 1 above in ‘Modelling abiotic factors’). The between-year variation in the seasonal pattern of roadkill (total abundance of all taxa combined) was not significantly associated with either temperature or rainfall (Table 1, ‘all taxa’). However, for specific taxa, some differences between years were explained at least in part by abiotic factors (Table 1). Temperature did not have a significant effect on any taxa. Significantly fewer roadkill were associated with high rainfall for three of the eighteen taxa: gulls (Chi sq. = 12.003, d.f. = 1, *p* < 0.001), magpies (Chi sq. = 9.173, d.f. = 1, *p* = 0.002), and rabbits (Chi sq. = 4.37, d.f. = 1, *p* = 0.037).

## Discussion

This study reveals substantial seasonal variation in the occurrence of wildlife roadkill in the UK across multiple taxa, and taxon-specific differences in the nature of that seasonal variation. Across taxa, two broad temporal patterns of seasonality were found: bimodal or unimodal. Where seasonality was present (i.e. for all taxa except foxes), peaks in roadkill reports are likely linked to periods of increased activity, such as mate-searching, foraging, feeding young, and dispersal [11, 31, 48]. Understanding these patterns can provide valuable insights into the behaviour and ecology of the taxa recorded (and by extension, that of scavengers using roadkill as a food resource), as well as opportunities to implement effective mitigation on roads to reduce wildlife mortality, and the risk of injury to drivers as a result of wildlife-vehicle collision [11]. The use of temporary warning signs, for example, has been shown to reduce deer collisions by 50% [49], and temporary drift fences in hotspots of chelonian roadkill have reduced roadkill by 98% [50]. There is limited evidence to assess the long-term effectiveness of permanent animal warning signs [51], but temporary signs can help to overcome the habituation effect of ‘sign-blindness’ that occurs when signs are used long-term. In addition to intrinsic biotic drivers of temporal wildlife roadkill, we find that between-year patterns for some taxa can be affected by abiotic factors (temperature and rainfall); notably, high rainfall was associated with reduced road mortality for 3 of the 19 taxa (gulls, magpies, and rabbits).

### Temporal patterns across all taxa

The broad summer mortality pattern of total roadkill could be due to breeding activity in temperate regions, where many taxa produce young during the spring and early summer [48, 52] and therefore by late summer population sizes are high, boosted by the abundance of young of the year. Not only does this mean more individuals, so increasing the probability of a vehicle collision, but also an abundance of inexperienced juveniles which must disperse and may not yet have learned to avoid vehicles. Birds, for example, learn to adjust their flight initiation distance depending on the traffic speed [19], a skill that recently fledged birds are likely to lack. Further evidence that juveniles may be more vulnerable to vehicle collisions comes from captive-bred (and therefore road naïve) Tasmanian devils (*Sarcophilus harrisii*), which when released to the wild are more likely to be struck by vehicles than their wild-bred counterparts [53]. In addition, some adult animals may also be more vulnerable to vehicle collisions at this time as parents will be actively provisioning dependent juveniles, requiring more frequent or longer foraging trips [17], which may increase the frequency with which they encounter roads.

While animal behaviour likely underlies many of the temporal patterns observed here, abiotic factors also played a role for some, but not all taxa. Rainfall had a significant effect, and was associated with reduced roadkill of gulls, magpies, and rabbits. We suggest this is likely due to their reduced activity during rainfall [11] but cannot rule out some degree of bias (e.g. a reduction in reporting during wet weather due to reduced visibility). However, if this were the main driver, we would expect similar impacts for other taxa, which is not apparent.

#### Bimodal seasonal patterns

Bimodality was the most common temporal pattern across all taxa, although the peak in observations were not coincidental across taxa in the months in which they occurred. Wildlife-vehicle collisions are strongly correlated with the number of road crossings made [17], so increased movement or population size might contribute to seasonal peaks. In the current study the bimodal patterns are likely to arise as a result of increased activity and large-scale movement related to breeding for the first peak, followed by juvenile dispersal for the second peak, and the troughs a function of reduced activity levels, e.g. as food becomes scarce in winter [54]. Consistent with this interpretation, roadkill polecats are mainly adult males in spring and juveniles in autumn [55]. Badger roadkill usually peaks in February-April, as has been widely observed by others [2, 17, 29, 48] and again in July to September [48]. Although badgers are able to mate throughout the year, breeding occurs primarily in March-April and, to a lesser extent, in autumn [56]. For roe deer the pattern is similar, with a peak in March-May and a second small peak in autumn, highlighting two time-periods of a potentially greater risk of motorists becoming involved in serious accidents. The dispersal of young male roe deer in spring, and the territorial and reproductive behaviour of adult males during spring and summer, could explain the increase in roadkill during this season [57, 58]. Surprisingly, our data did not show a second peak in July-August (as has been demonstrated in other studies, and linked to the rutting season [e.g. 59]. In England, at least 6,000 geo-referenced occurrences of deer vehicle collisions were reported between 2008–2014 [24]; identifying seasonal peaks in deer activity on roads is therefore extremely important with respect to focused periods for mitigation.

Seasonal peaks in brown rats and pheasants are likely driven by anthropogenic activities. Rats are capable of breeding throughout the year if conditions are suitable [60], and it is therefore unlikely that the consistent September peak was solely due to mate-searching or juvenile dispersal. The majority of arable farms in the UK complete their harvest by the end of September [61], which we hypothesise will remove an important source of cover and food for this species, subsequently leading to increased levels of movement, and increased contact with roads. For pheasants, the autumn peak coincides with the release of captive-bred birds onto shoots, but the highest peak in pheasant roadkill is in the spring. In line with previous studies, the large spring peak is likely caused by cessation of supplementary feeding, leading to increased foraging movement by pheasants [18].

#### Unimodal seasonal patterns

Unimodal peaks in roadkill were more commonly observed for birds than mammals, and the timing of the peak within the year was taxon dependent. In mammals, the time period between adult breeding, and juvenile dispersal, can lead to two distinct peaks in roadkill; in birds, the time period between these events is usually much shorter [62]. The temporal convergence of mate searching, provisioning of young, and juvenile dispersal could therefore explain the common unimodal mortality patterns seen in birds. Four of the eight bird taxa examined here (gulls, woodpigeons, magpies and blackbirds) demonstrated a single consistent peak in roadkill numbers centred around the summer months, contrasting with the weak patterns shown by the birds of prey, and the strongly bimodal peaks of pheasants. Gull chicks usually hatch in June in the UK [63], and many are unable to fly when they leave the nest in July, consistent with the peak in roadkill observations. Although many pigeon species can breed all year round, the main nesting season for the woodpigeon is between March and September, with peak egg-laying taking place in April and May [52], such that fledging pigeons may explain the single seasonal peak centred on July. Similarly, magpies peaked in June and July, at a time when magpie chicks usually fledge [64]. Blackbirds have a more variable, protracted and less prominent peak than most of the other birds, likely due to their extended breeding season; they can successfully rear two or more broods in a year depending on weather conditions [65].

For mammals that followed a unimodal pattern in roadkill, mate searching may not play a large role in road-associated mortality risk. Juvenile dispersal likely represents the most parsimonious explanation for the unimodal summer peak in roadkill mortality in rabbits, which can reach high densities near roads due to both the abundance of suitable vegetation and ’predator release’ resulting from road avoidance by predators during the day when disturbance from vehicles is highest [66–68]. Otters showed a single peak during the winter months—the only mammals in our study to do so. In the UK, river flow is generally higher in winter than in summer [69]. Otters may be unable or unwilling to swim through structures such as bridges and culverts during periods of high flow and so be forced to cross roads to continue their journeys [3]. Indeed, 65% of otter roadkill mortalities occur within 100m of a watercourse, and of these, around 34% occur at bridges and 44% at culverts [70].

Foraging can increase movement of animals, and so increase the probability of vehicular collision. Hedgehog roadkill reports peaked in July and August of each year, and was lowest in the winter months, as has been found in other countries (Italy: [71]; Poland: [72]). Hedgehogs are most active during the summer [73], when they may travel several kilometres in a night [74] in search of food—roadkill reports are highest around July, when juveniles begin to leave the nest [75]. Similarly, foraging may be the underlying cause of the September peak in grey squirrels. This is the time of year when squirrels perform food-caching behaviour, ready for consumption in the winter [76], and is the time of highest population densities; females can produce one or two litters of pups per year, in the spring (March/April) and summer (July/August) [76].

#### No seasonality

The red fox and Reeves’ muntjac deer lacked seasonal peaks. This observation for red foxes contradicts studies from other countries; for example, in Portugal, where spring and early summer are peak periods for red fox road mortality, and one study recording 42% of the entire annual mortality between May and July [17] when juveniles disperse [77]. We propose that the lack of seasonality could reflect the road-adapted nature of this species in the UK. The red fox is well adapted to and found in high population densities in urban areas in many parts of the UK [78, 79] and the species has acquired ‘road sense’ in urban areas [77]. The consistent availability of carcasses along roadsides likely contributes to the presence of foxes near roads throughout the year [12]. Urban foxes in the UK have previously been found to scavenge more frequently in winter [78], potentially leading to more road crossings (and mortality) at this time. Either adaptation might mask a potential summer mortality peak associated with seasonal dispersal.

The lack of seasonality in Reeves’ muntjac deer roadkill reports is unsurprising because, unlike other deer species, muntjac breed throughout the year and do not have distinct seasonal reproductive peaks [80]. With births being equally spread throughout the year, the distribution of naïve young, potentially more at risk from WVC, will be fairly even across the months. Additionally, the lack of a distinct rutting season reduces the likelihood of a seasonal peak in roadkill male muntjac deer [80].

The absence of significant seasonality for the predatory birds (barn owl, buzzard and tawny owl), and for polecats may reflect a true lack of seasonality in road mortality risk for these species. It is notable that all are predatory (or scavengers). Year-round availability of prey on roads may contribute to a seasonality in roadkill of these species [13]. It is also notable, however, that (with the exception of the fox) those species showing no significant seasonality are also the least well represented in terms of sample size.

### Citizen science as a tool for roadkill monitoring

The present study highlights the potential of citizen science data to contribute to our understanding of wildlife-human interactions on a temporal scale. There are, however, obvious limitations that come with using *ad hoc* data, including heterogenous survey effort in temporal and spatial contexts [81], and the mixed reliability of species detectability and identification [82, 83], that must be acknowledged when utilising these data. We have attempted to deal with these issues by, for instance, requiring a minimum number of reports per species to be included in analysis, the conversion of absolute abundances into monthly proportional data, and the acknowledgement of limitations associated with *ad hoc* data collection. Similarly, species misidentification is also a potential limitation for a number of other highly valued long-term volunteer monitoring programmes that take place in the UK, including the Big Garden Birdwatch (https://www.rspb.org.uk/get-involved/activities/birdwatch/), Breeding Bird Survey (https://www.bto.org/our-science/projects/bbs) and the Biological Records Centre (https://www.brc.ac.uk/). Resources like the National Biodiversity Network also rely upon accumulating *ad hoc* wildlife sightings from various sources across the UK yet prove a valuable resource with over 235 million wildlife records to date (https://nbn.org.uk/). The future of citizen science for roadkill monitoring is likely to see a greater emphasis on the standardization of data collection and validation [84], along with the introduction of identification tools or resources for volunteers, as have already been adopted by some studies [85, 86]. The rapid removal of roadkill by scavengers also contributes to underreporting of carcasses, and although estimations of carcass persistence have been attempted [87–89] this is another potential source of bias. The trade-off between data quality and quantity is an important consideration, and retaining volunteer interest and participation is key to the success of these programmes.

### Implications and applications

Although the biomass of roadkill was not estimated from this study, it clearly represents a substantial, year-round resource that can sustain an artificially high density of meso-predators and scavengers. Although scavengers may provide an important ecosystem service by removing carcasses from the environment [13, 90], there may also be negative impacts, such as increased predation on eggs or chicks of native birds when the relative abundance of roadkill versus live prey shifts [10]. The presence of roadkill in an environment may also create a ‘landscape of fear’, in which predators attracted by the carcasses lead to avoidance of the area by smaller prey species, such as rodents [91].

Reducing wildlife-vehicle collisions is clearly desirable in terms of wild animal welfare and conservation, but it would also pay dividends in terms of improved safety for vehicle drivers. The insights provided by this study could provide ways of minimising roadkill, for example by installing temporary road warning signage at times of greatest risk for car drivers, and at roadkill blackspots for species of conservation concern (e.g. otters). Here, through long term monitoring of wildlife roadkill, we provide an insight into temporal patterns of key UK taxa. Using citizen science data in this way, and applying it to on-the-ground mitigation, could lead to both better protection for species of conservation concern, and a reduced risk of human fatalities and injuries on roads. The citizen science approach has huge future potential to provide insights into the true extent of WVC on a large scale, especially with the incorporation of a wider variety of temporal predictors, greater volunteer education and potential standardization of different roadkill projects across the globe. This approach will be particularly useful in countries or regions, or for species, where other sources of monitoring data (such as direct observation or hunting records) are not available.

## Supporting information

S1 Text Summary of reporting information. (DOCX)

S1 Fig Patterns in reporting habits. Produced using recorderMetrics package in R [51]. (a) Number of active days—the number of individual days that each reporter contributed on, (b) Activity Ratio—the proportion of active days to number of days a volunteer was linked to the project, (c) the total number of days volunteers participated and (d) the standard deviation of time between each pair of active days per volunteer.
(TIF)

S2 Fig Seasonal patterns in reporters. The number of reporters per month displayed as a percentage of the total number of reporters for that year. All six years are shown.
(TIF)

S3 Fig Seasonal patterns in records. The number of roadkill records per month as a percentage of the overall number of records for that year. Data from 2014–2019 are shown.
(TIF)
