# Correction: Temporal patterns of wildlife roadkill in the UK

**DOI:** 10.1371/journal.pone.0310789

**Published:** 2024-09-16

**Authors:** Sarah Raymond, Amy L. W. Schwartz, Robert J. Thomas, Elizabeth Chadwick, Sarah E. Perkins

In Table 1, the shading of data cells was incorrectly applied. Cells shaded in grey should correspond to significant p-values (P<0.05); however, this was not consistently observed. Please see the corrected Table 1 here.

**Table 1:** Summary table of statistical values (Chi sq. and p-value) for Generalised Additive Models examining relationships between seasonal roadkill patterns and two abiotic variables; mean temperature and rainfall. Statistically significant values are shaded. In all instances, the degrees of freedom were 1.

Taxon	Mean Temperature		Rainfall		Model family
	Chi. sq	p-value	Chi. Sq	p-value	
All taxa	0.072	0.789	1.14	0.286	Poisson
Badgers	1.014	0.314	0.202	0.653	Poisson
Foxes	0.328	0.567	1.627	0.202	Poisson
Otters	1.753	0.186	0.612	0.434	Poisson
Polecats	1.798	0.18	0.034	0.853	Neg. binomial
Brown Rats	0.001	0.973	0.54	0.817	Poisson
Grey Squirrels	0.015	0.902	1.797	0.18	Poisson
Hares	3.064	0.08	0.518	0.472	Poisson
Rabbits	0.4	0.527	4.37	0.037	Poisson
Hedgehogs	0.004	0.949	0.767	0.381	Poisson
Barn Owls	0.019	0.891	0.449	0.503	Neg. binomial
Tawny Owls	0.227	0.633	1.452	0.228	Neg. binomial
Buzzards	0.236	0.627	0.038	0.846	Neg. binomial
Pheasants	0.419	0.517	0.12	0.729	Poisson
Gulls	2.931	0.087	12.003	<0.001	Poisson
Woodpigeons	0.055	0.815	0.195	0.659	Poisson
Magpies	0.041	0.839	9.173	0.002	Neg. binomial
Blackbirds	0.199	0.656	2.899	0.089	Neg. binomial
Muntjac Deer	0.22	0.639	0.269	0.604	Neg. binomial
Roe Deer	0.206	0.65	0.044	0.833	Neg. binomial

## References

[1] Raymond S, Schwartz ALW, Thomas RJ, Chadwick E, Perkins SE (2021) Temporal patterns of wildlife roadkill in the UK. PLOS ONE 16(10): e0258083. 10.1371/journal.pone.0258083 34613989 PMC8494347

