# Supplementary material for: Temporal patterns of wildlife roadkill in the UK
Source: PLoS One. 2021 Oct 6;16(10):e0258083. doi: 10.1371/journal.pone.0258083 (PMC8494347; doi:10.1371/journal.pone.0258083)
Supplement: S1 Text — (DOCX) [file pone.0258083.s001.docx]

**S1 Text. Summary of reporting information**

The mean number of reports per year was 9040 (±1455) and the mean number of individual reporters per year was 562 (±105). Across the six-year period of this study, there were a total of 2672 contributors and 54,240 individual roadkill records. A large proportion of reporters (n=1178, 44%) reported only once (S1 Fig), while >80% of reporters contributed on less than 10 occasions over 6 years. The majority of participants contributed for a short period of time and largely, if they contributed more than once, there was little variation in the time between sequential reports (S1 Fig; [1]).

During July 2019, Project Splatter took part in a major press release, resulting in a sharp increase in the number of contributors and records for the following two months (S2 and S3 Figs). This spike in interest was the basis for randomly subsampling the July and August datasets from 2019 to get an adjusted mean number of each species, as outlined in the main manuscript.

**References:**

1. August T, Fox R, Roy DB, Pocock MJO. Data-derived metrics describing the behaviour of field-based citizen scientists provide insights for project design and modelling bias. Scientific Reports. 2020;10(1):1–12.
