# Supplementary figures and images for: Temporal patterns of wildlife roadkill in the UK
Source: PLoS One. 2021 Oct 6;16(10):e0258083. doi: 10.1371/journal.pone.0258083 (PMC8494347; doi:10.1371/journal.pone.0258083)

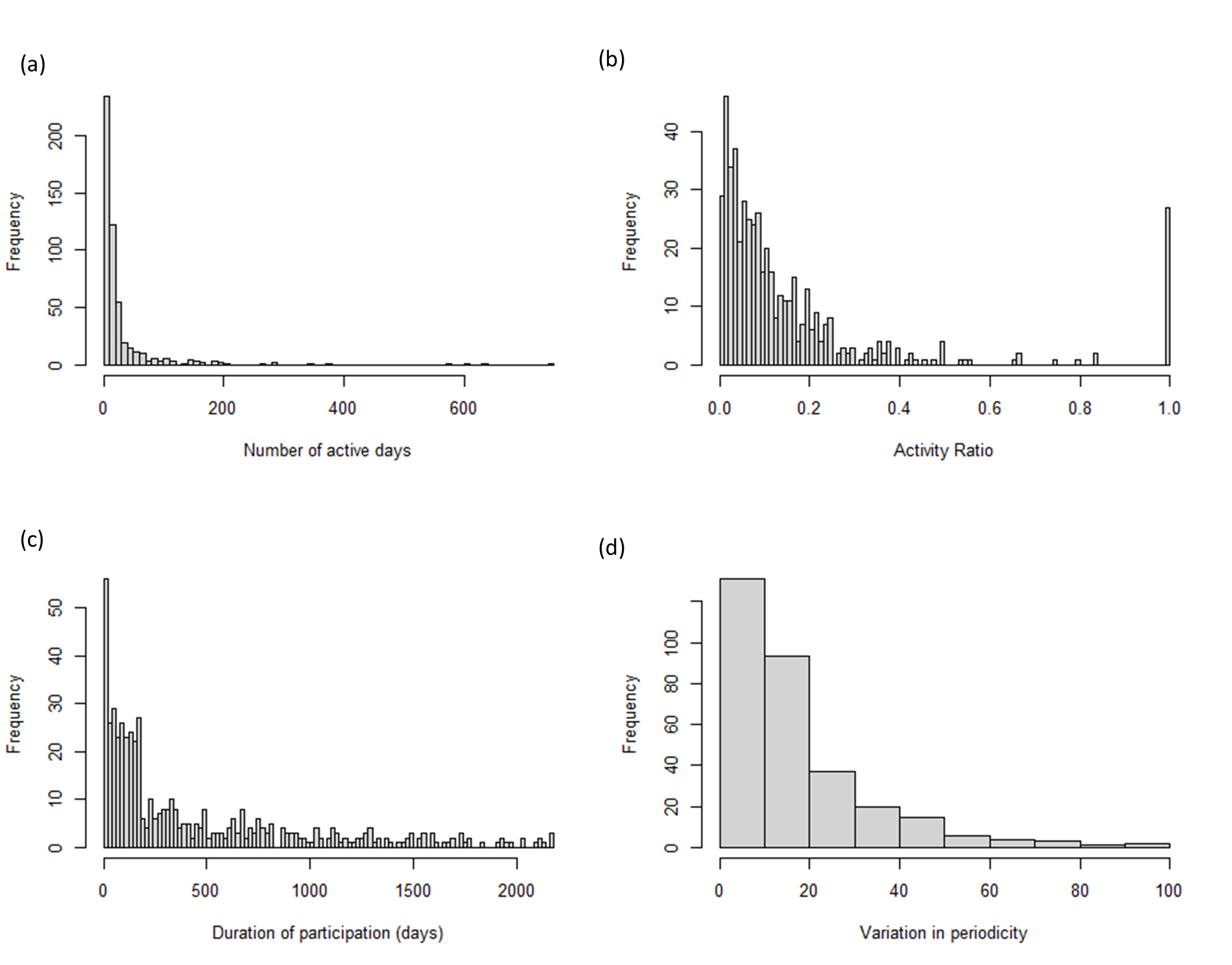

Supplement: S1 Fig — Produced using recorderMetrics package in R [51]. (a) Number of active days—the number of individual days that each reporter contributed on, (b) Activity Ratio—the proportion of active days to number of days a volunteer was linked to the project, (c) the total number of days volunteers participated and (d) the standard deviation of time between each pair of active days per volunteer. (TIF) [file pone.0258083.s002.tif]

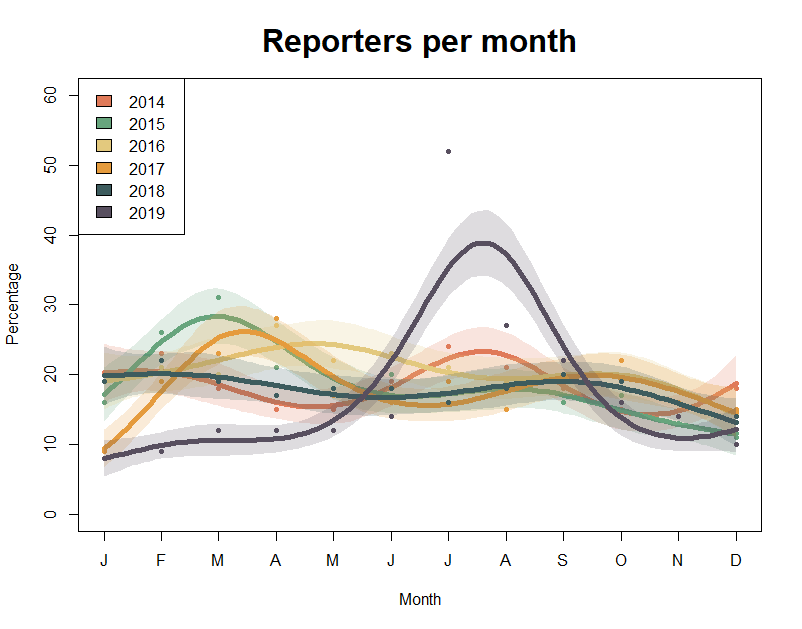

Supplement: S2 Fig — The number of reporters per month displayed as a percentage of the total number of reporters for that year. All six years are shown. (TIF) [file pone.0258083.s003.tif]

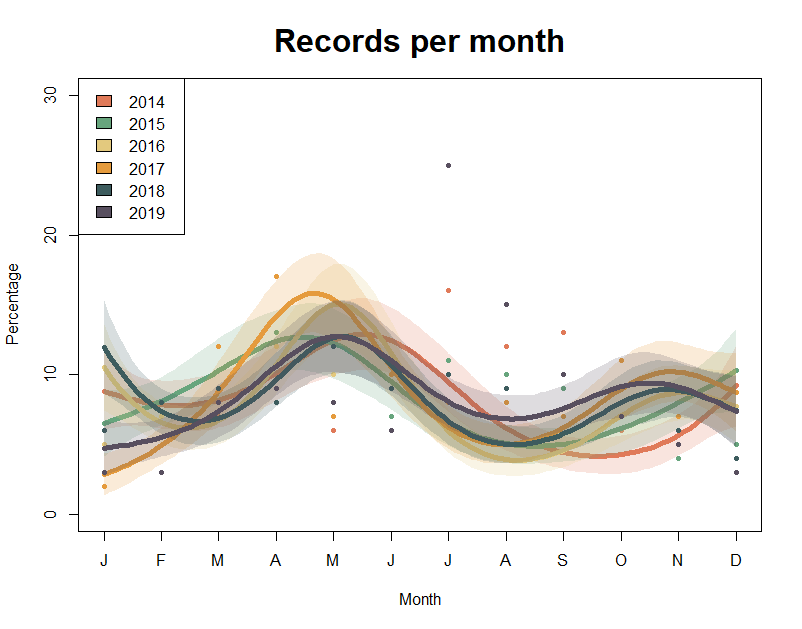

Supplement: S3 Fig — The number of roadkill records per month as a percentage of the overall number of records for that year. Data from 2014–2019 are shown. (TIF) [file pone.0258083.s004.tif]
